# Pressure dispersion pad use allows patients to kneel comfortably after total knee arthroplasty

**DOI:** 10.1002/jeo2.70157

**Published:** 2025-04-24

**Authors:** Kunihiko Watamori, Kazunori Hino, Tatsuhiko Kutsuna, Tomofumi Kinoshita, Takashi Tsuda, Hiromasa Miura, Masaki Takao

**Affiliations:** ^1^ Department of Orthopaedic Surgery Ehime University Graduate School of Medicine Matsuyama Japan; ^2^ Department of Orthopaedic Surgery Kyusyu Rosai Hospital Kitakyushu Japan

**Keywords:** contact pressure, kneeling, pressure dispersion pad, total knee arthroplasty

## Abstract

**Purpose:**

This study aimed to clarify the contact pressure at which patients with difficulty in kneeling after total knee arthroplasty (TKA) feel pain and the contact pressure at which kneeling can be performed after using a pressure dispersion pad.

**Methods:**

Sixty patients (69 knees) who underwent TKA for end‐stage knee osteoarthritis were included. The patients performed single stance kneeling on the sheet‐type pressure mapping system, and the contact pressure and area were measured before and after using the pressure dispersion pad. The Oxford Knee Score was used to evaluate kneeling ability.

**Results:**

The group of patients who were unable to kneel had significantly lower contact pressure than those who were able to kneel easily (0.61 N/cm^2^/kg vs. 0.99 N/cm^2^/kg; *p* = 0.04). No patient reported kneeling as being ‘impossible’ or ‘extremely difficult’ when using the pressure dispersion pad. Moreover, all patients except those without pain had less pain and improved kneeling scores when using the pressure dispersion pad. Use of the pressure dispersion pad significantly reduced contact pressure for all kneeling score groups after TKA (0.12 N/cm^2^/kg for the impossible group).

**Conclusions:**

The patients who could not kneel after TKA felt pain at 61% of the contact pressure compared to those who could kneel easily. Even patients who were unable to kneel after TKA were able to kneel when the contact pressure was reduced to 12% of that of the patients who could easily kneel using a pressure dispersion pad.

**Level of Evidence:**

Level III.

**Abbreviations**
BMI: body mass index
BW: body weight
load/BW: total load applied
NRS: Numerical Rating Scale
OKS: Oxford Knee Score
TKA: total knee arthroplasty

## INTRODUCTION

Total knee arthroplasty (TKA) is a reliable surgical procedure for reconstructing impaired knee joints. TKA can improve pain relief, functional mobility and patients' quality of life [7, 22, 31]. However, 11%–25% of postoperative TKA patients are not satisfied with their surgery [4, 6]. One of the reasons is the difficulty in performing several activities of daily living after TKA, contrary to patients' expectations. Kneeling is important for many daily activities and holds significant cultural, religious, and occupational values for patients [10]. In a previous study, 52% of patients who underwent TKA reported kneeling as the most important activity involving the affected knee. Meanwhile, 72% of the patients reported kneeling as their most difficult activity after TKA [35]. The inability to kneel affects many daily and leisure activities such as cleaning, gardening, and sports, and consequently has been reported to negatively impact the emotional well‐being, social independence and overall happiness of patients who undergo TKA [10]. Therefore, we considered it necessary to focus on improving the ability to kneel. A previous study reported a strong correlation between kneeling and knee pain [36].

Various factors cause pain during kneeling [11, 23, 27, 32, 36]; several studies have reported that the overlap between the contact area and incision site during kneeling is strongly correlated with pain [11, 17, 23, 24, 32]. To improve kneeling in patients after TKA, it is important to control the mechanical load applied to the incision site during kneeling. However, the contact pressure at which a patient feels pain after TKA, and the extent to which this pressure can be lowered to enable kneeling, remain unclear.

We hypothesized that in patients who are unable to kneel due to pain after TKA, reducing contact pressure to below the individual patient's pain threshold using a pressure dispersion pad will allow the patient to kneel and improve the quality of life.

The purposes of this study were to clarify the contact pressure at which patients who have difficulty kneeling after TKA feel pain and the contact pressure at which kneeling becomes possible using a pressure dispersion pad.

## METHODS

### Participants' characteristics

Overall, 60 patients (69 knees) underwent primary TKA for end‐stage knee osteoarthritis. We excluded patients with post‐traumatic osteoarthritis, rheumatoid arthritis and diseases of other joints or the spine. The patients comprised 43 women (48 knees) and 17 men (21 knees). The timing of evaluation in this study was 3 years (range, 1–6 years) after TKA. A board‐certified orthopaedic knee surgeon, with experience in over 3000 TKA cases, performed the procedures in all patients. A midline skin incision with a medial parapatellar approach was used for each TKA procedure. Regarding the surgical methods in TKA, the mechanical alignment and measured resection technique were utilized in this study. To determine the femoral component's rotational angle, we used the surgical epicondylar axis as the index of femoral rotation. Before surgery, we calculated the angle gap between the surgical epicondylar axis and the posterior condylar axis on the axial view of computed tomography to determine the rotational angle. The tibial rotational axis was set parallel to the line connecting one‐third of the tibial tubercle to the centre of the cut surface. Soft tissues were balanced to achieve varus‐valgus stability. Twenty‐six patients (28 knees) received a cruciate‐retaining knee prosthesis (MERA Quest Knee System, Senko Medical Instrument Manufacturing Co. Ltd.) and 34 patients (41 knees) received a posterior‐stabilized knee prosthesis (NexGen LPS‐Flex, Zimmer). The patella was resurfaced, using a cemented polyethylene button, in all 69 knees. All patients underwent the standardized postoperative rehabilitation regimen, and kneeling was allowed at 3 months post‐TKA. Since the contact pressure and contact area during kneeling of healthy individuals had also not been reported previously, 26 healthy men and women were recruited to a volunteer group as controls. The control group included 13 women (13 knees) and 13 men (18 knees).

The study was approved by the institutional review board of our university's graduate school of medicine (identification number: 1709019). All participants provided written informed consent.

### Measurement and evaluation methods

During the measurement of contact pressure and contact area, the ability to kneel was assessed using a question from the Oxford Knee Score (OKS) [9]: ‘Can you kneel down?’ Patients' answers and scores are shown in Table 1. This score has been widely used in previous studies [3, 13, 34]. Subsequently, we used the Numerical Rating Scale (NRS) to evaluate kneeling pain.

**Table 1:** Kneeling score: Answers to the question ‘Can you kneel down?’.

Yes, easily	4
With little difficulty	3
With moderate difficulty	2
With extreme difficulty	1
No, impossible	0

*Note*: Patients were surveyed on their ability to kneel and were asked to choose one of the following five categories: no difficulty, little difficulty, moderate difficulty, extreme difficulty or unable to kneel.

There are two versions of the NRS: NRS‐11 and NRS‐101. In this study, we used NRS‐11 because it allows for easier scoring. A score of 0 represents ‘no pain’, and a score of 10 represents ‘the worst possible pain’ or ‘the most intense pain imaginable’. A previous systematic review reported that the NRS showed higher compliance and ease of use compared to the visual analogue scale [14].

**Figure 1:**
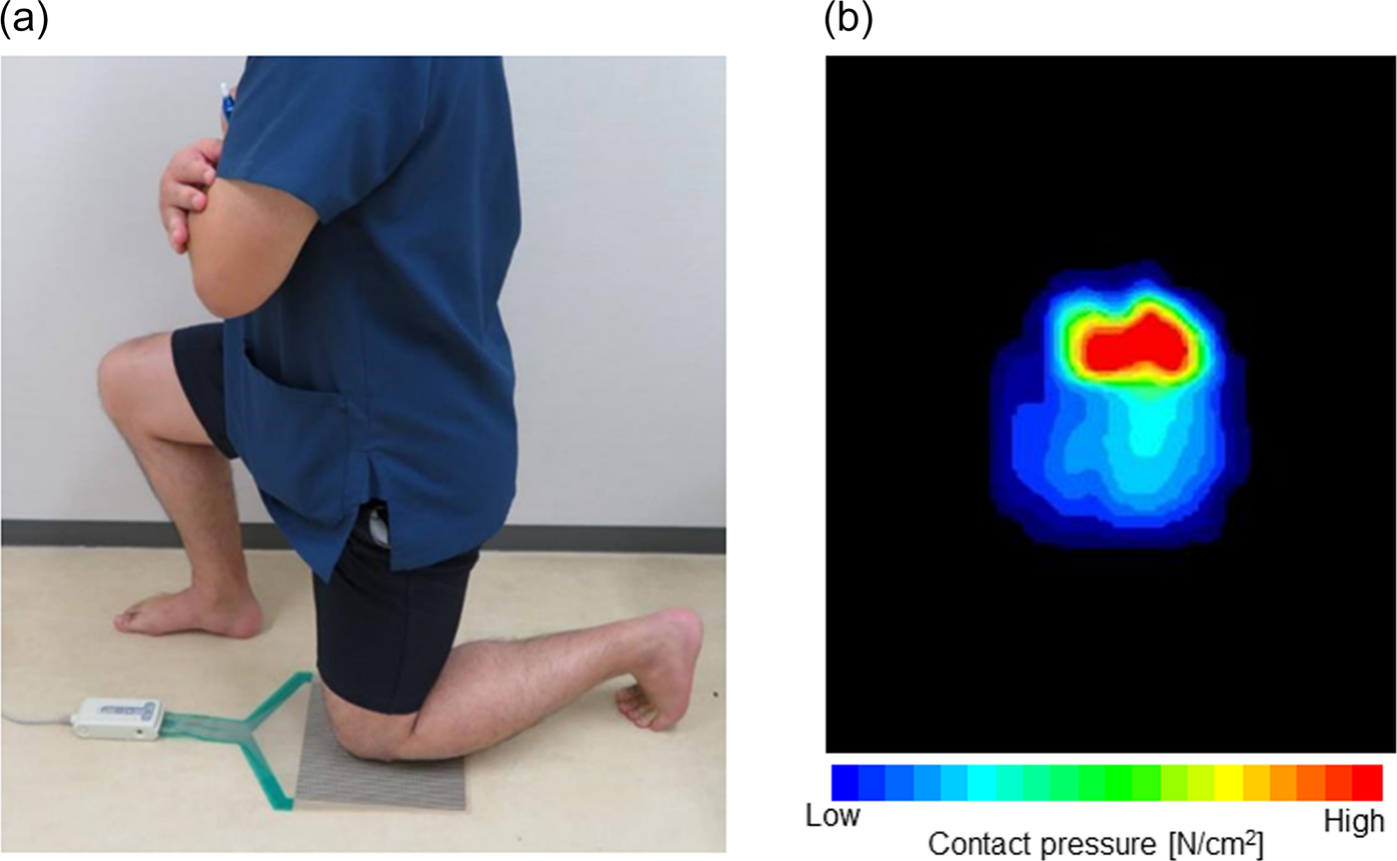
Measurement of the contact pressure using the sheet‐type pressure mapping system. (a) Each participant was asked to face forward while kneeling for 10 s on the sensor. (b) A colour map showing the distribution of contact pressure around the anterior knee during kneeling.

Contact pressure and contact area were measured using a sheet‐type pressure mapping system (I‐Scan, TekScan). The plastic‐laminated thin film (0.1 mm thick) has an electronic pressure sensor with two 9.2‐cm^2^ sensing arrays, each with 2288 sensing elements (Figure 1). This film has previously been used in other studies to measure contact surface areas and peak pressures [28, 33]. During measurements, patients were instructed to apply as much load as possible to the kneeling side. Patients who answered that they were unable to perform kneeling were assisted until they assumed the kneeling position but were not assisted during measurement. Each participant was asked to face forward while kneeling, for 10 s, at 90° knee flexion without using hand support. We measured the contact pressure, total contact area, and load of the kneeling side as the participants knelt directly on the hard floor (Figure 1). To calculate the load applied when kneeling, we divided the total load by the total body weight (load/BW). We conducted the measurement three times and calculated the average value. The measurements were made by an orthopaedic knee surgeon. Next, the patient placed the decompression pad we developed between the sensor and the knee, and the same measurements were performed again. The questionnaire was administered again to evaluate changes in kneeling ability and pain.

### Development of the pressure dispersion pad

We used a 3D printer to create a patella model with a diameter of 80 mm that was then attached to the STB‐1225S testing device (A&D Co. Ltd.) and pressurized to create an environment similar to kneeling (Online Resource 1). The materials used for cushioning were six types of polyurethane foam with different coefficients of repulsion used in neck collars and corsets in the orthopaedic field (Online Resource 2).

The thickness of the materials was 10 mm, and a 30‐mm pad was created by layering three layers of the same material or two layers of one material with another material sandwiched between these two layers. All combinations were subjected to the aforementioned compression testing machine at a speed of 20 mm/min and a maximum load of 600 N. The average value was calculated after repeating the pressure test three times. Since the combination of the two materials was able to distribute the load more efficiently than a single material, the contact pressure and contact area were measured only for the combination of the two materials (Online Resource 3). It was noted that the combination of urethane foams with different coefficients of restitution and sandwiching a material with a low coefficient of restitution with a material with a high coefficient of restitution resulted in the lowest contact pressure. In this study, the pressure dispersion pad was made with the combination of No. 6 for the first and third layers and No. 2 for the second layer, which decreased the contact pressure and increased the contact area the most (Online Resource 4). The rebound resilience of materials No. 6 and No. 2 was 46% and 4%, respectively.

### Statistical analyses

Statistical analyses were performed using the JMP 14 software system (SAS Institute Inc.). Data are expressed as median and interquartile range. A nonparametric test was employed because normal distribution could not be assumed for some patient groups. Comparisons of paired continuous variables were performed using the Wilcoxon signed‐rank test. Comparisons of continuous variables between multiple groups were performed using the Steel–Dwass test. *p* values < 0.05 were considered statistically significant. Power and Sample Size Calculations software version 3.1.2 (Vanderbilt University) was used to calculate the number of samples required to detect changes in contact pressure before and after the use of the pressure dispersion pad during kneeling in post‐TKA patients. The number of samples required to detect changes in contact pressure before and after the use of a pressure dispersion pad during kneeling for a post‐TKA patient was calculated. Since there were no previous data on contact pressure during kneeling in post‐TKA patients, a power analysis was performed based on the study data of 10 post‐TKA patients. The average value of the difference before and after using the pad was 0.4 N/kg/cm^2^, and the standard deviation was 0.4 N/kg/cm^2^. The sample size that could detect an average contact pressure difference of 0.4 N/cm^2^/kg with a Type 1 error probability of 0.05 and a detection power of 0.8 was 10.

## RESULTS

The participants' characteristics are shown in Table 2.

**Table 2:** Participants' characteristics.

Variable	Volunteer group (*n* = 30)	TKA group (*n* = 60)	*p* value	Kneeling score					*p* value
				0	1	2	3	4	
Age (years), mean (SD)	25.8 ± 7.0	74.7 ± 6.8	<0.01*	73.4 ± 8.2	75.6 ± 4.2	74.2 ± 6.3	74.2 ± 6.3	72.4 ± 8.2	0.57^§^
Males, *n*	17	21	0.01^†^	3	4	3	7	4	0.90**
Height (cm), mean (SD)	164.0 ± 7.9	155.2 ± 8.5	<0.01*	154.8 ± 8.1	154.4 ± 8.9	153.4 ± 1.0	156.0 ± 8.6	157.7 ± 7.5	0.68^§^
Weight (kg), mean (SD)	60.2 ± 12.2	62.3 ± 9.5	0.35*	60.9 ± 8.4	66.2 ± 11.4	59.8 ± 8.7	62.8 ± 9.2	62.2 ± 10.5	0.48^§^
BMI (kg/m^2^), mean (SD)	22.1 ± 2.9	25.8 ± 2.4	<0.01*	25.3 ± 1.5	27.6 ± 2.5	25.4 ± 2.2	25.8 ± 2.7	24.8 ± 2.3	0.06^§^

*Note*: Values are presented as counts (*n*) and proportions (%) for male sex. Age, height, weight and BMI are presented as mean ± SD. Between‐group differences were analyzed using the *Mann–Whitney test, ^†^Chi‐square test, ^§^Kruskal–Wallis test and **Fisher's Exact Probability test.

Abbreviations: BMI, body mass index; SD, standard deviation; TKA, total knee arthroplasty.

### Contact pressure before using a pad

**Figure 2:**
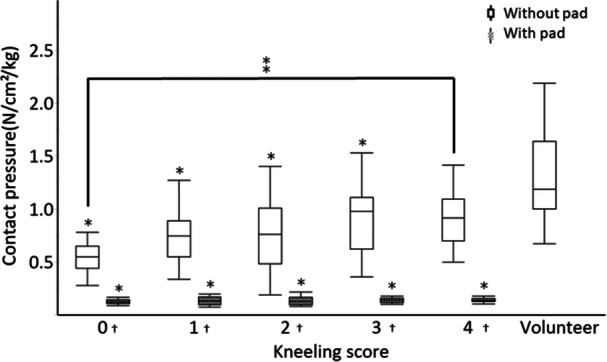
Contact pressure during kneeling with and without pressure dispersion pads for each kneeling score group. The graph shows the contact pressure during kneeling with and without a pressure dispersion pad for each kneeling score group. The volunteer group was only evaluated without pressure dispersion pads. The horizontal line shows each kneeling score and volunteer, and the vertical line shows the contact pressure (N/cm^2^/kg). ⁎, The score 0, 1, 2 and 3 groups had significantly lower contact pressures than those of the volunteer group (*p* < 0.05); †, all score groups had significantly lower contact pressures when pressure dispersion pads were used (*p* < 0.05); ⁑, the score 0 group had a significantly lower contact pressure before using the pad compared to the score 4 group (*p* < 0.05).

**Figure 3:**
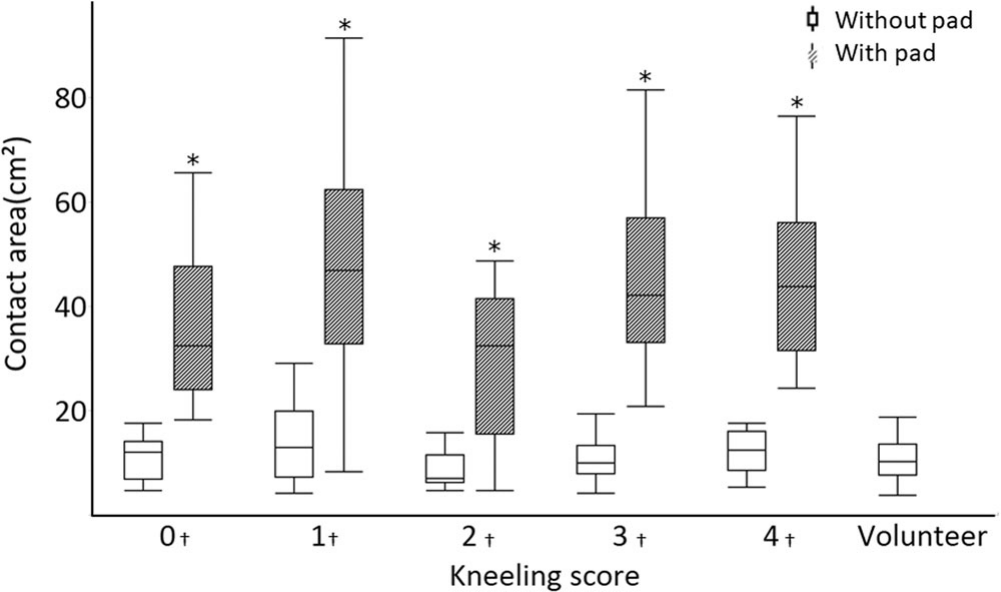
Contact area during kneeling with and without pads for each kneeling score group and volunteers. The graph shows the contact area during kneeling with and without pressure dispersion pads for each kneeling score group. The volunteer group was only evaluated without pressure dispersion pads. The horizontal line shows each kneeling score and volunteer, and the vertical line shows the contact area (cm^2^). ⁎, Using a pressure dispersion pad, the contact area increased more for all score groups than for the volunteer group (*p* < 0.05); †, all score groups showed an increased contact area when pressure dispersion pads were used (*p* < 0.05).

**Figure 4:**
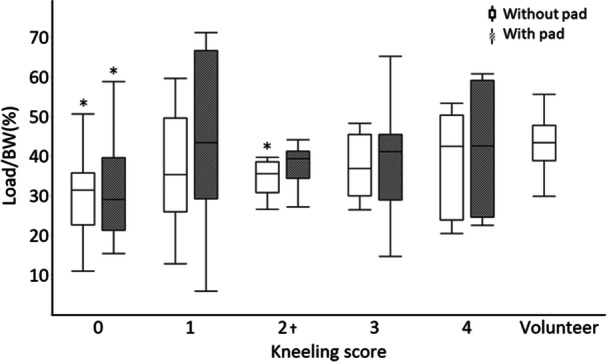
Load/body weight (BW) during kneeling with and without pads for each kneeling score group and volunteers. The graph shows the load/BW during kneeling with and without pressure dispersion pads for each kneeling score group. The volunteer group was only evaluated without pressure dispersion pads. The horizontal line shows each kneeling score and volunteer, and the vertical line shows the load/body weight (%). ⁎, Without pressure dispersion pads, the score 0 and 2 groups had a significantly lower load/body weight than the volunteer group (*p* < 0.05). With pressure dispersion pads, the score 0 group had a significantly lower load/BW than the volunteer group (*p* < 0.05); †, the score 2 group had a significantly lower load/BW when pressure dispersion pads were used (*p* < 0.05).

Kneeling scores after TKA were as follows: 18 knees (26%) could not kneel and kneeling was extremely difficult for 13 knees (19%), moderately difficult for 12 knees (18%), a little difficult for 16 knees (23%) and easily performed for 10 knees (14%) (Table 3). Regarding contact pressure, it was significantly lower in the impossible group than in the no‐difficulty group (*p* = 0.04) (Figure 2). The average contact pressure of the impossible group was 61% of that of the no‐difficulty group. Contact area and load/BW were not significantly different between the groups (Figures 3 and 4).

**Table 3:** Improvement of kneeling score with use of decompression pad.

		Kneeling score without pad					Total
		0	1	2	3	4	
Kneeling score with pad	**2**		3	1			4
	**3**	10	7	8	5		30
	**4**	8	3	3	11	10	35
	**Total**	18	13	12	16	10	69

**Figure 5:**
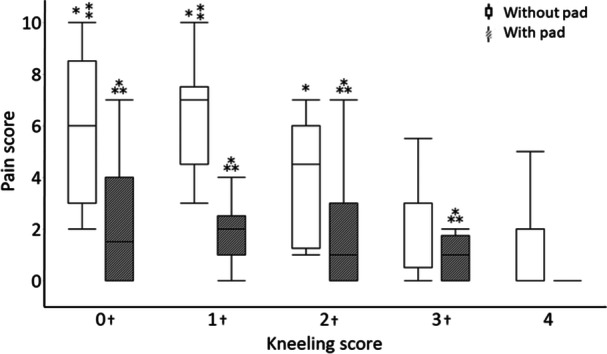
Pain score during kneeling with and without pads for each kneeling score. The graph shows the pain score during kneeling with and without pressure dispersion pads of each kneeling score group. The horizontal line shows each kneeling score, and the vertical line shows the pain scores. ⁎, Without pressure dispersion pads, the score 0, 1 and 2 groups had a significantly higher pain score than the score 4 group (*p* < 0.05); ⁑, Without pressure dispersion pads, the score 0 and 1 groups had a significantly higher pain score than the score 3 group (*p* < 0.05); ⁂, With pressure dispersion pads, the score 0, 1, 2 and 3 groups had a significantly higher pain score than the score 4 group (*p* < 0.05); †, The pain score decreased for the score 0, 1, 2 and 3 groups when pressure dispersion pads were used (*p* < 0.05).

Pain scores were significantly higher in the impossible, extremely difficult, and moderately difficult groups than in the no‐difficulty group (*p* < 0.01, *p* < 0.01 and *p* = 0.02, respectively). The pain scores of the impossible and extremely difficult groups were significantly higher than those of the little difficulty group (*p* = 0.01 and *p* = 0.02, respectively) (Figure 5).

When compared with the normal knees of the control group, the contact pressure in each kneeling score‐based group, except for the easy group, was significantly lower than that in the control group (Table 4, Figure 2). Regarding the contact area, there was no significant difference between the control group and each kneeling score‐based group after TKA (Table 4, Figure 3), and the load/BW was significantly lower in the impossible and moderately difficult groups than in the control group (Table 4, Figure 4).

**Table 4:** Comparison of contact pressure, area and load/BW for each kneeling score group and volunteers.

	Kneeling score	Without pad	With pad
Contact pressure	0	<0.01*	<0.01*
	1	<0.01*	<0.01*
	2	<0.01*	<0.01*
	3	0.03*	<0.01*
	4	0.16	<0.01*
Contact area	0	1.00	<0.01*
	1	0.95	<0.01*
	2	0.32	0.01*
	3	1.00	<0.01*
	4	0.82	<0.01*
Load/BW	0	<0.01*	<0.01*
	1	0.49	1.00
	2	<0.01*	0.54
	3	0.22	0.7
	4	0.99	1.00

*Note*: Steel–Dwass test; load/BW, amount of load on the kneeling side/body weight.

* <0.05.

### Contact pressure after using a pressure dispersion pad

No patient reported kneeling as being ‘impossible’ or ‘extremely difficult’ when using the pad. Moderate difficulty was reported for four knees (6%), little difficulty for 30 knees (44%) and easy for 35 knees (50%), indicating improvement in all patients. The contact pressure significantly decreased after pad use in each kneeling score‐based group while the contact area significantly increased (Figures 2 and 3, Table 5). The contact pressure was reduced to 12%–14% of that of the easy group with the use of the pad. A comparison of load/BW before and after using the pad revealed a significant difference only in the moderate difficulty group (Figure 4, Table 5). Pain scores improved significantly before and after pad use in the impossible, extremely difficult, moderately difficult and little difficult groups (Figure 5, Table 5). There were no significant differences in contact pressure, contact area and load/BW between the groups when using a pad (Figures 2, 3, 4).

**Table 5:** Comparison of variables during kneeling with and without pads use by kneeling score group.

		Volunteer	Patient				
			Kneeling score				
			0	1	2	3	4
Pain score	Without pad		6.0 (3.0–8.5)	7.0 (4.5–7.5)	4.5 (1.25–6.0)	3.0 (0.5–3.0)	0.0 (0–2)
	With pad		1.5 (0–4.0)	2.0 (1.0–2.5)	1.0 (0–3.0)	1.0 (0–1.75)	0 (0–0)
	*p*		<0.01*	<0.01*	<0.01*	<0.01*	0.25
Contact pressure (N/cm^2^/kg)	Without pad	1.18 (1–1.64)	0.55 (0.44–0.65)	0.75 (0.54–0.88)	0.76 (0.48–1.00)	0.97 (0.62–1.11)	0.91 (0.70–1.1)
	With pad		0.12 (0.11–0.14)	0.13 (0.1–0.17)	0.12 (0.1–0.16)	0.13 (0.11–0.16)	0.13 (0.12–0.15)
	*p*		<0.01*	<0.01*	<0.01*	<0.01*	<0.01*
Contact area (cm^2^)	Without pad	10.24 (7.61–13.54)	12.0 (6.88–14.05)	12.88 (7.17–19.90)	6.99 (6.22–11.49)	9.99 (7.90–13.31)	12.44 (8.52–15.95)
	With pad		32.34 (24.00–47.56)	46.83 (32.78–62.35)	32.34 (15.44–41.42)	42.00 (33.00–56.93)	43.60 (31.54–55.98)
	*p*		<0.01*	<0.01*	<0.01*	<0.01*	<0.01*
Load/BW (%)	Without pad	43.39 (38.82–47.72)	31.35 (22.61–35.71)	35.33 (25.96–49.52)	35.49 (30.72–38.47)	36.88 (29.91–45.40)	42.45 (23.85–50.30)
	With pad		29.03 (21.23–39.59)	43.32 (29.18–66.63)	39.36 (34.33–41.21)	41.06 (28.88–45.41)	42.55 (24.54–59.06)
	*p*		0.70	0.16	0.02*	0.50	0.16

*Note*: Wilcoxon signed‐rank test; load/BW, amount of load on the kneeling side/body weight.

* <0.05.

## DISCUSSION

In this study, contact pressure on the anterior aspect of the knee during kneeling was measured in patients after TKA according to their kneeling scores. We also examined how much contact pressure can be reduced by using a pressure dispersion pad to improve the kneeling ability. There are two new findings of this study. First, the contact pressure on the anterior aspect of the knee during kneeling in patients who were unable to kneel after TKA was 61% of the contact pressure in patients who were able to kneel. Second, even patients who were unable to kneel after TKA could kneel if the contact pressure was reduced to 12 % of that of the difficulty group. To our knowledge, no previous study has quantitatively measured the contact pressure during kneeling in patients after TKA. Goldstein et al. investigated the contact pressure during kneeling using the Apex Harris Mat (Aetrex) and tactile pressure‐indicating film (Sensor Products) [11] and reported the contact area, but did not quantitatively measure the contact pressure.

The quantitative measurement of contact pressure during kneeling in this study revealed that the contact pressure of patients who were unable to kneel was lower than that of normal individuals and TKA patients who could kneel. One possible reason for the higher pain score despite the lower contact pressure is a decrease in pain threshold among TKA patients. Previous studies have reported that patients who experience pain during activities after TKA have lower pain tolerance than patients without pain; however, the reason for lower pain thresholds after TKA remains unclear [2, 20, 38]. These studies have reported that post‐TKA patients with low pain thresholds have more intense pain at rest, during walking, and at night. Kneeling is more tasking than walking or resting. A previous study showed that patellofemoral contact pressure during kneeling increased significantly compared to when the knee is unloaded or during squatting [21]. A past study examining kneeling also reported pain as the main reason for not being able to kneel upright [5]. Moreover, almost all patients had difficulty kneeling on the side of the knee after TKA [5]. This result suggests that many patients may be able to kneel if their pain is reduced.

The main solution to pain during kneeling, as reported in several past studies, is to avoid overlapping the area of load concentration and the incision site [11, 17, 24, 32]. In this study, we used a pressure dispersion pad to prevent the load from concentrating on a specific location and to reduce pain. Using a pressure dispersion pad significantly increased the contact area and decreased the contact pressure during kneeling. As a result, pain was reduced in 98% of the patients, except for eight patients who originally had no pain, and the kneeling score improved in 96% of the patients. Similarly, the use of pressure dispersion pads to disperse contact pressure and reduce pain has been reported in a study on chronic plantar tendonitis [8]. The advantage of this method is that it can be adapted to all patients and may improve activities of daily living in patients who were unsatisfied after TKA because of the inability to kneel. We assumed that the use of pressure dispersion pads would increase the load/BW during kneeling, but the results were contrary to our hypothesis. The load/BW increased significantly after pad use only in the moderate difficulty group, but no significant differences were noted in the other patient groups. Furthermore, there was no significant difference in load after use of the pressure dispersion pad except in the moderate difficulty group. This indicates that the ease of kneeling for patients after TKA is determined by the contact pressure and not by the amount of load. Differences in thresholds of pain in response to contact pressure might be associated with the ease of postoperative kneeling in TKA patients. Factors that may contribute to differences in pain thresholds include incision site [17, 24, 32], saphenous neuropathy [24], preoperative or perioperative pain sensitization [20, 29, 38] and patient‐specific pain thresholds [20, 38], which require further investigation.

Other patient‐related factors that may influence kneeling, include sex [15, 26], body mass index (BMI) [26] and knee flexion [37]. Regarding sex, a report states that women have a significantly higher kneeling ability [15], while another self‐reported study suggests that men have a significantly higher kneeling ability [26]. Additionally, it has been reported that the OKS used to evaluate kneeling ability is significantly lower in women [12]. We analyzed the contact pressure, contact area and load/BW for only the female participants, but the results remained unchanged (Online Resources 5 and 6). Regarding BMI, a previous study indicated that patients with BMIs above 33 kg/m^2^ have a significantly lower likelihood of being able to kneel [26]. In this study, a significant difference in preoperative BMI was observed between the patient and volunteer groups, but no significant differences were found among the patient groups for each kneeling score, indicating that preoperative BMI did not influence kneeling ability. Regarding knee flexion, some studies indicate that there is no association with kneeling ability [3, 16, 25]. Meanwhile, one study suggests a significant relationship between postoperative knee flexion and kneeling ability [37]. In this study, the preoperative and postoperative knee flexion for TKA patients were 122° and 126°, respectively. Since there were no significant differences in knee flexion among the various kneeling score groups, it can be concluded that knee flexion did not influence kneeling ability.

This study has some limitations. First, we did not measure the contact pressure and load during kneeling before surgery. Second, we evaluated a single design of a pressure dispersion pad in this study. The average contact pressure at which patients who could not kneel were able to kneel after pad use was 0.13 N/cm^2^/kg; however, if various designs of pads are used, it may be possible to kneel at a higher contact pressure. To resolve these limitations, additional studies with a larger sample size are necessary. However, reducing the contact pressure to 12%–14% of that of the difficulty group using the pad reduced the pain during kneeling in most patients, thereby improving the post‐TKA kneeling ability. Third, this study included patients who underwent bilateral TKA. Conditions may differ between patients who underwent unilateral and bilateral TKA. In this study, we evaluated kneeling using single‐stance kneeling rather than double‐stance kneeling, assessing contact pressure separately for each side, which included bilateral cases. However, since bilateral TKA might interfere, we evaluated only the joints with unilateral TKA, and similar results were observed (Online Resources 7 and 8). Fourth, this study did not verify whether the use of pad improved patient satisfaction after TKA. Kneeling is an important activity in daily life; [10] the factors that influence satisfaction after TKA are multifactorial, with kneeling being only one [19, 30]. The necessity of kneeling varies among individuals and has been reported to differ based on factors such as occupation and religion [1, 6, 18, 19]. We believe that improving satisfaction with TKA requires an approach tailored to individual needs.

## CONCLUSIONS

Patients who were unable to kneel after TKA experienced pain at 61% of the contact pressure at knee contact of patients who were able to kneel without problems. Further, even patients who were unable to kneel after TKA could kneel if the contact pressure was reduced to 12% of that of easy group using a pressure dispersion pad.

## AUTHOR CONTRIBUTIONS

**Kunihiko Watamori**: Conceptualization; methodology; formal analysis; investigation; writing—original draft preparation; writing—review and editing. **Kazunori Hino**: Conceptualization; methodology; formal analysis; investigation; writing—review and editing. **Tatsuhiko Kutsuna**: Conceptualization; formal analysis; investigation; writing—review and editing. **Tomofumi Kinoshita**: Conceptualization; formal analysis; investigation; writing—review and editing. **Takashi Tsuda**: Conceptualization; formal analysis; investigation; writing—review and editing. **Hiromasa Miura**: Conceptualization; methodology; writing—review and editing. **Masaki Takao**: Writing—review and editing; supervision.

## CONFLICT OF INTEREST STATEMENT

The authors declare no conflicts of interest.

## ETHICS STATEMENT

All procedures performed in studies involving human participants were in accordance with the ethical standards of the institutional and/or national research committee and with the 1964 Helsinki Declaration and its later amendments or comparable ethical standards. The study was approved by the institutional review board of our university's graduate school of medicine (identification number: 1709019). Informed consent was obtained from all individual participants included in the study.

## Supporting information


**Online Resource 1.** Compression testing set‐up for development of a pressure dispersion pad. The figure shows (a) Overview of compression testing machine (STB‐1225S), (b) A patellar‐like metal and tested pad on the compression sensor.

**Online Resource 2.** Types of urethane or polyethylene pad with different properties and combination methods. The figure shows (a) six types of urethane or polyethylene foams with different coefficients of repulsion used in neck collars and corsets in the orthopedic field, (b) how the pads are made. The thickness of the materials was 10 mm, and a 30‐mm pad was created by layering three layers of the same material or two layers of one material with another material sandwiched between these two layers.

**Online Resource 3.** Color map of compression pressure of each combination of urethane pads. The figure shows the contact pressure and contact area when each pad is used. The numbers under the color map indicate the number of the material used.

**Online Resource 4.** The average contact pressure of each combination of pads. The figure shows the change in contact pressure when a force of 600 N is applied at 20 mm/s using each pad. The horizontal line shows each combination of pad, and the vertical line shows the contact pressure. The contact pressure decreased the most when the No. 6 material was used on the outside and the No. 2 material was used on the inside.

Supporting information.

## Data Availability

The data are available from the corresponding author on reasonable request.
