# Supplementary figures and images for: Pressure dispersion pad use allows patients to kneel comfortably after total knee arthroplasty
Source: J Exp Orthop. 2025 Apr 24;12(2):e70157. doi: 10.1002/jeo2.70157 (PMC12019298; doi:10.1002/jeo2.70157)

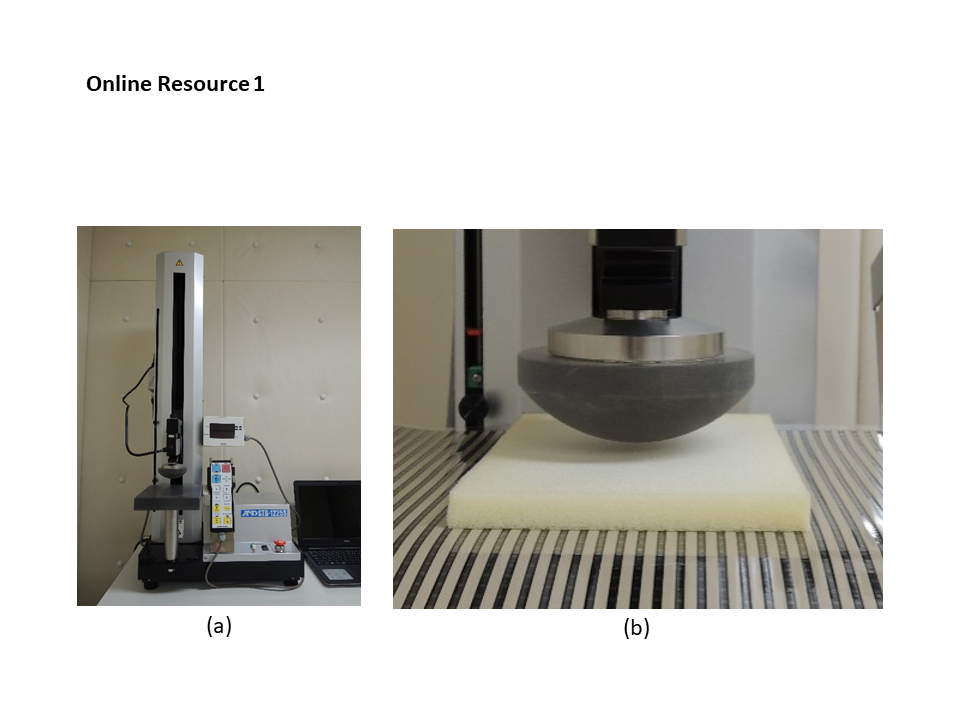

Supplement: Supplementary file 1 — Online Resource 1. Compression testing set‐up for development of a pressure dispersion pad. The figure shows (a) Overview of compression testing machine (STB‐1225S), (b) A patellar‐like metal and tested pad on the compression sensor. [file JEO2-12-e70157-s001.TIF]

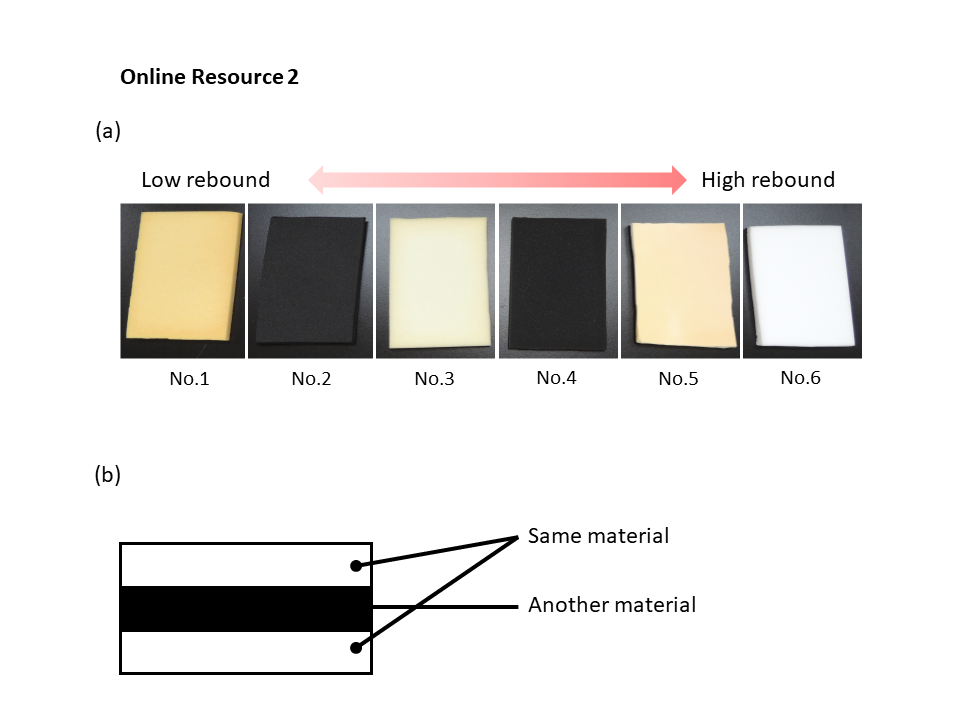

Supplement: Supplementary file 2 — Online Resource 2. Types of urethane or polyethylene pad with different properties and combination methods. The figure shows (a) six types of urethane or polyethylene foams with different coefficients of repulsion used in neck collars and corsets in the orthopedic field, (b) how the pads are made. The thickness of the materials was 10 mm, and a 30‐mm pad was created by layering three layers of the same material or two layers of one material with another material sandwiched between these two layers. [file JEO2-12-e70157-s004.TIF]

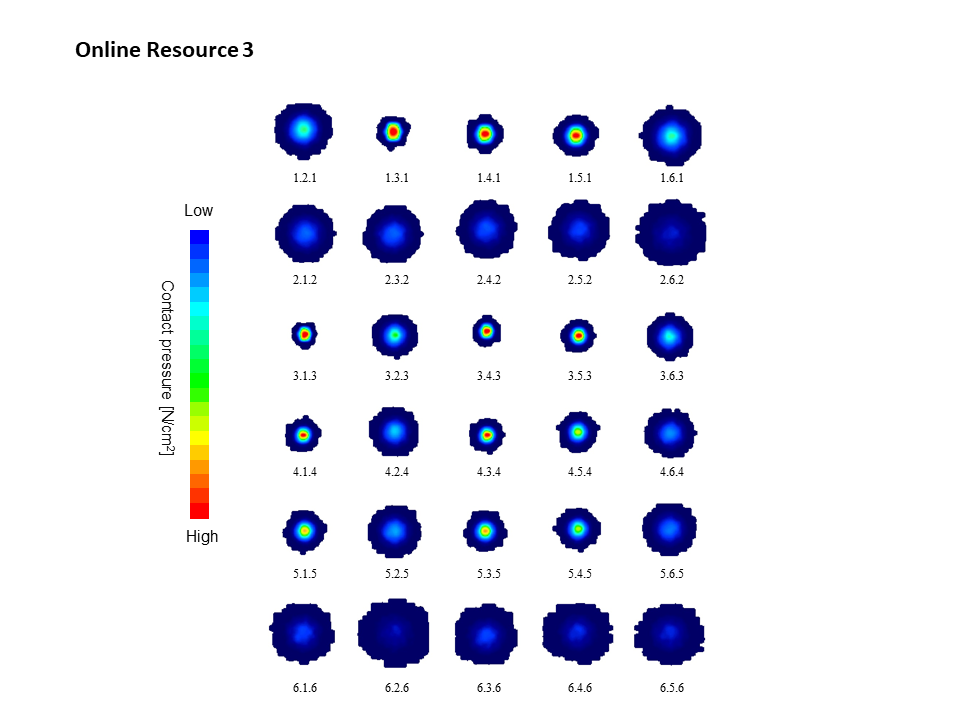

Supplement: Supplementary file 3 — Online Resource 3. Color map of compression pressure of each combination of urethane pads. The figure shows the contact pressure and contact area when each pad is used. The numbers under the color map indicate the number of the material used. [file JEO2-12-e70157-s005.TIF]

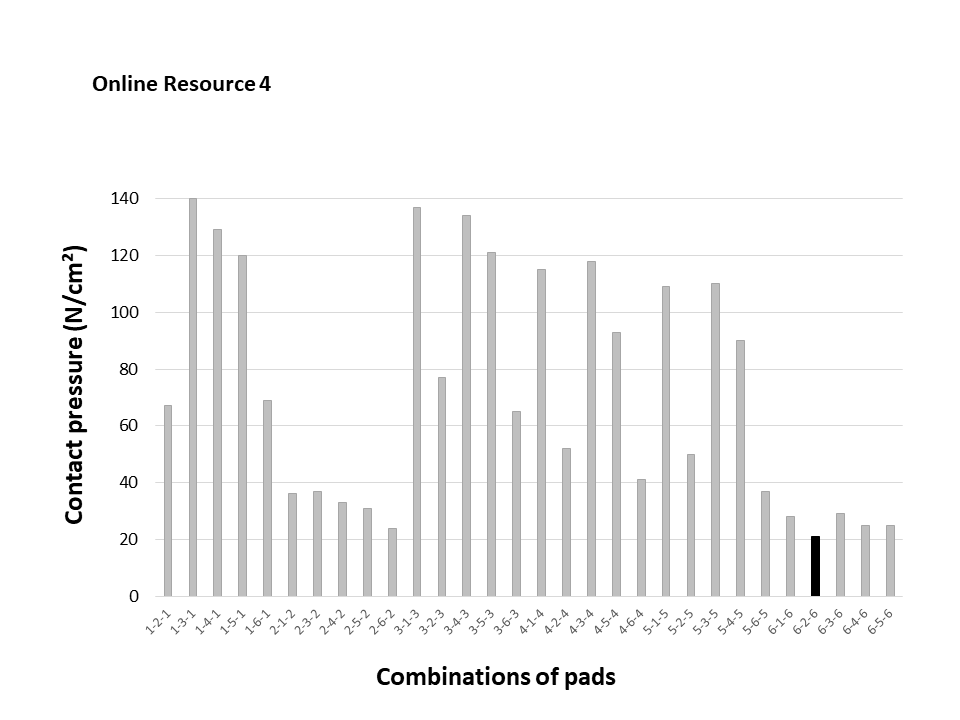

Supplement: Supplementary file 4 — Online Resource 4. The average contact pressure of each combination of pads. The figure shows the change in contact pressure when a force of 600 N is applied at 20 mm/s using each pad. The horizontal line shows each combination of pad, and the vertical line shows the contact pressure. The contact pressure decreased the most when the No. 6 material was used on the outside and the No. 2 material was used on the inside. [file JEO2-12-e70157-s003.TIF]
