# Supplementary material for: Pressure dispersion pad use allows patients to kneel comfortably after total knee arthroplasty
Source: J Exp Orthop. 2025 Apr 24;12(2):e70157. doi: 10.1002/jeo2.70157 (PMC12019298; doi:10.1002/jeo2.70157)
Supplement: Supplementary file 5 — Supporting information. [file JEO2-12-e70157-s002.docx]

**Pressure Dispersion Pad Use Allows Patients to Kneel Comfortably after Total Knee Arthroplasty**

The Journal of Experimental Orthopaedics

Watamori K^1^; Hino K^1^, PH.D.; Kutsuna T^1^; Kinoshita T^1^; Tsuda T^1^; Miura H^2^; Takao M^1^*

^1^ Department of Orthopaedic Surgery, Ehime University Graduate School of Medicine

^2^ Department of Orthopaedic Surgery, Kyusyu Rosai Hospital

***Corresponding author**

Masaki Takao

E-mail: [takao.masaki.ti@ehime-u.ac.jp](mailto:takao.masaki.ti@ehime-u.ac.jp)

Ehime University Graduate School of Medicine, Shitsukawa, Toon, Ehime, JP 791-0295, +81-89-960-5343

Fax: +81-89-960-5346

**Online Resource 5**. **Comparison of contact pressure, area, and load/BW by kneeling score group for women only**

|  | Kneeling score | Without pad | With pad |
| --- | --- | --- | --- |
| Contact pressure | 0 | <0.01* | <0.01* |
|  | 1 | <0.01* | <0.01* |
|  | 2 | <0.01* | <0.01* |
|  | 3 | 0.03* | <0.01* |
|  | 4 | 0.58 | <0.01* |
| Contact area | 0 | 0.71 | <0.01* |
|  | 1 | 0.48 | <0.01* |
|  | 2 | 1.00 | 0.03* |
|  | 3 | 0.89 | <0.01* |
|  | 4 | 0.28 | <0.01* |
| Load/BW | 0 | 0.01* | 0.02* |
|  | 1 | 0.89 | 0.99 |
|  | 2 | 0.05* | 0.42 |
|  | 3 | 0.13 | 0.51 |
|  | 4 | 0.13 | 1.00 |

Steel–Dwass test,　* <0.05. *Load/BW*, amount of load on the kneeling side/body weight

**Online Resource 6**. **Comparison of variables during kneeling with and without pad by kneeling score group for women**

|  |  | Volunteer | Patient | | | | |
| --- | --- | --- | --- | --- | --- | --- | --- |
|  |  |  | Kneeling score | | | | |
|  |  |  | 0 | 1 | 2 | 3 | 4 |
| Pain score | Without Pad |  | 6.0 (3.0-8.5) | 7.0 (4.5-7.5) | 4.5 (1.25-6.0) | 3.0 (0.5-3.0) | 0.0 (0-2) |
|  | With Pad |  | 1.5 (0-4.0) | 2.0 (1.0-2.5) | 1.0 (0-3.0) | 1.0 (0-1.75) | 0 (0-0) |
|  | p-value |  | <0.01* | <0.01* | <0.01* | 0.02* | 1.00 |
| Contact pressure (N/cm²/kg) | Without Pad | 1.63 (1.19-1.80) | 0.55 (0.44-0.65) | 0.75 (0.54-0.88) | 0.76 (0.48-1.00) | 0.97 (0.62-1.11) | 0.91 (0.70-1.1) |
|  | With Pad |  | 0.12 (0.11-0.14) | 0.13 (0.1-0.17) | 0.12 (0.1-0.16) | 0.13 (0.11-0.16) | 0.13 (0.12-0.15) |
|  | p-value |  | <0.01* | <0.01* | <0.01* | <0.01* | 0.03* |
| Contact area (cm²) | Without Pad | 7.61 (5.56-8.78) | 12.0 (6.88-14.05) | 12.88 (7.17-19.90) | 6.99 (6.22-11.49) | 9.99 (7.90-13.31) | 12.44 (8.52-15.95) |
|  | With Pad |  | 32.34 (24.00-47.56) | 46.83 (32.78-62.35) | 32.34 (15.44-41.42) | 42.00 (33.00-56.93) | 43.60(31.54-55.98) |
|  | p-value |  | <0.01* | <0.01* | <0.01* | <0.01* | 0.03* |
| Load/ BW (%) | Without Pad | 43.98 (37.61-46.15) | 31.35 (22.61-35.71) | 35.33 (25.96-49.52) | 35.49 (30.72-38.47) | 36.88 (29.91-45.40) | 42.45 (23.85-50.30) |
|  | With Pad |  | 29.03 (21.23-39.59) | 43.32 (29.18-66.63) | 39.36 (34.33-41.21) | 41.06 (28.88-45.41) | 42.55 (24.54-59.06) |
|  | p-value |  | 1.00 | 0.10 | 0.04* | 0.43 | 0.09 |

Wilcoxon signed-rank test, * <0.05

*Load/BW*, amount of load on the kneeling side/body weight

**Online Resource 7**. **Comparison of variables by kneeling score group for patients who underwent unilateral TKA only**

|  | Kneeling score | Without pad | With pad |
| --- | --- | --- | --- |
| Contact pressure | 0 | <0.01* | <0.01* |
|  | 1 | <0.01* | <0.01* |
|  | 2 | 0.04* | <0.01* |
|  | 3 | 0.05* | <0.01* |
|  | 4 | 0.16 | <0.01* |
| Contact area | 0 | 0.98 | <0.01* |
|  | 1 | 1.00 | <0.01* |
|  | 2 | 0.28 | 0.02* |
|  | 3 | 0.91 | <0.01* |
|  | 4 | 0.82 | <0.01* |
| Load/BW | 0 | <0.01* | 0.03* |
|  | 1 | 0.33 | 0.97 |
|  | 2 | 0.02* | 0.50 |
|  | 3 | 0.12 | 0.63 |
|  | 4 | 0.99 | 1.00 |

Steel–Dwass test,　* <0.05.

*Load/BW*, amount of load on the kneeling side/body weight; *TKA*, total knee arthroplasty

**Online Resource 8**. **Comparison of variables during kneeling with and without pad by kneeling score for unilateral TKA**

|  |  | Volunteer | Patient | | | | |
| --- | --- | --- | --- | --- | --- | --- | --- |
|  |  |  | Kneeling score | | | | |
|  |  |  | 0 | 1 | 2 | 3 | 4 |
| Pain score | Without Pad |  | 5.0 (3.0-9.5) | 5.0 (4.0-8.0) | 5.5 (3.5-6.75) | 4.0 (2.75-6.38) | 0.0 (0-2.0) |
|  | With Pad |  | 2.0 (0.13-4.0) | 2.0 (2.0-3.0) | 2.0 (0-3.0) | 1.0 (0.75-4.63) | 0 (0-0) |
|  | p-value |  | <0.01* | <0.01* | 0.02* | 0.03* | 0.25 |
| Contact pressure (N/cm²/kg) | Without Pad | 1.18 (1-1.64) | 0.55 (0.44-0.63) | 0.81 (0.60-0.92) | 0.81 (0.48-1.05) | 1.22 (0.75-1.51) | 0.91 (0.70-1.10) |
|  | With Pad |  | 0.12 (0.11-0.14) | 0.12 (0.09-0.17) | 0.12 (0.10-0.17) | 0.13 (0.11-0.17) | 0.13 (0.12-0.15) |
|  | p-value |  | <0.01* | <0.01* | <0.01* | 0.03* | <0.01* |
| Contact area (cm²) | Without Pad | 10.24 (7.61-13.54) | 12.73 (7.68-14.05) | 11.12 (6.73-13.17) | 6.85 (6.22-8.56) | 8.49 (5.17-11.58) | 12.44 (8.52-15.95) |
|  | With Pad |  | 34.54 (26.34-47.86) | 41.56 (31.03-60.30) | 32.34 (19.90-41.42) | 39.81 (21.66-49.76) | 43.76 (31.54-55.98) |
|  | p-value |  | <0.01* | <0.01* | <0.01* | 0.03* | <0.01* |
| Load/ BW (%) | Without Pad | 43.39 (38.82-47.72) | 31.35 (23.86-34.97) | 35.33 (23.30-48.73) | 35.09 (27.41-38.35) | 34.14 (27.26-40.73) | 42.45 (23.85-50.30) |
|  | With Pad |  | 30.88 (22.97-40.15) | 41.48 (21.33-46.41) | 39.49 (29.46-43.23) | 33.96 (24.31-50.82) | 42.55 (24.54-59.06) |
|  | p-value |  | 0.56 | 0.41 | 0.05* | 0.84 | 0.16 |

Wilcoxon signed-rank test, * <0.05

*Load/BW*, amount of load on the kneeling side/body weight; *TKA*, total knee arthroplasty
